# INTRAVENOUS IMMUNOGLOBULIN IN DERMATOLOGY

**DOI:** 10.4103/0019-5154.48996

**Published:** 2009

**Authors:** Sandipan Dhar

**Affiliations:** *From the Institute of Child Health and AMRI Hospitals, Kolkata, India.*

## Introduction

The intravenous administration of exogenous pooled human immunoglobulin (intravenous immunoglobulin — i.v. IG) was originally licensed as antibody replacement therapy in patients with primary immunodeficiencies. However, subsequently it has been successfully used in a host of diseases not amenable to other modalities of treatment. There are currently six FDA-approved uses for this agent. Despite a current lack of FDA approval, off-label treatment of a multitude of dermatologic disorders with i.v. IG has shown exciting potential for this unique treatment modality.[1–3] The dermatoses successfully treated with i.v. IG include autoimmune bullous diseases, connective tissue diseases, vasculitis, Stevens-Johnson syndrome (SJS), toxic epidermal necrolysis (TEN) and infectious disorders (such as streptococcal toxic shock syndrome).[4] Currently the biggest drawback in the consideration of i.v. IG therapy in dermatologic disorders is the lack of randomized controlled trials (RCTs). Nevertheless, there is a significant body of evidence demonstrating the efficacy of i.v. IG in patients with skin diseases that are resistant to treatment with standard agents.[3–5]

## Composition of Intravenous Immunoglobulin

Intravenous immunoglobulin (i.v. IG) is a sterile highly purified IgG preparation made from pooled human plasma and typically contains more than 95% of unmodified IgG, which has functionally intact Fe-dependent effector functions and only trace amount of IgA or IgM. It is composed of polyspecific immunoglobulin molecules prepared by cold ethanol fractionation of pooled human sera harvested from thousands of donors. Several measures are used to ensure the safety of the product[4,5]:

1. Careful selection of donors
2. Screening of every donation for hepatitis B surface antigen, antihepatitis C virus antibodies, anti-HIV 1 and 2 antibodies, syphilis serology and normal liver function
3. Use of viral inactivation procedure in addition to the already high viral inactivation afforded by cold ethanol fractionation

## Mechanism of Action of High-Dose Intravenous Immunoglobulin

The mechanism by which high-dose intravenous immunoglobulin (hd i.v. IG) mediates anti-inflammatory activity is not well understood. These effects are mediated via the Fc portion of IgG or the antigen-binding site and the variable regions of the antibody molecule. Proposed mechanisms of action of hd i.v. IG include the following[5,6]:

1. Anti-idiotype interactions
2. Fc receptor modulation
3. Modulation of the production of cytokines and cytokine antagonists
4. Neutralization of causative microbe or toxin
5. Superantigen neutralization
6. Effects on complement — inhibition of complement-mediated damage
7. Acceleration of IgG catabolism

## Pharmacokinetics[4–6]

Peak serum concentrations occur immediately after intravenous injection and are dose related. Within 24 hours, up to 30% of the dose may be removed by distribution and catabolism. i.v. IG distributes itself throughout the intravascular (60%) and extravascular (40%) spaces, crosses the placenta and may be excreted into milk. Serum half-life is 3 to 5 weeks [Table 1].

**Table 1:** Indications for the use of high dose intravenous immunoglobulin

FDA approved
Kawasaki disease
Non-FDA approved
Connective tissue disease
Dermatomyositis
Autoimmune bullous disease
Pemphigus vulgaris and foliaceus
Bullous pemphigoid
Cicatricial pemphigoid
Herpes gestationis
Linear IgA disease
Epidermolysis bullosa acquisita
Hypersensitive dermatoses
Erythema multiforme
Toxic epidermal necrolysis
Chronic urticaria
Other dermatoses
Atopic dermatitis
Pyoderma gangrenosum
Scleromyxedema

## Autoimmune Bullous Diseases

High-dose i.v. IG is useful for patients when[6] —

1. Conventional therapy has failed
2. Significant adverse effects of conventional therapy exist
3. Absolute and relative contraindications to the use of high-dose long-term systemic steroids or immunosuppressive agents exist
4. Disease is progressive in spite of administering appropriate maximum yet safe conventional systemic therapy
5. Rapidly progressive epidermolysis bullosa acquisita (EBA) with generalized cutaneous disease exists

## Other Conditions Treated with hd i.v. IG

Dermatomyositis represents the most extensively studied dermatological condition with regards to hd i.v. IG therapy.[7] Généreau *et al.*[8] reported a case in which antimalarial-resistant cutaneous LE was treated successfully with hd i.v. IG. Prins *et al.*[9] and Trent *et al.*[10] reported beneficial effect of hd i.v. IG in TEN. Its beneficial effect has been documented in a number of reports across the globe.[11–13] The standardized mortality ratio (SMR) showed a trend to lower actual mortality with i.v. IG treatment as compared to the predicted mortality.[13] On the contrary, Bachot *et al.*[14] report ineffectiveness of hd i.v. IG in a study of 34 patients with TEN. Wolff *et al.*[15] in their editorial comments try to explain this divergent conclusion as batch-to-batch variation in the capacity of hd i.v. IG to inhibit FAS-mediated cell death.

## Dose

A dose of 1-2 g/kg is recommended, usually delivered as a 5-consecutive-day cycle of 0.4 g/kg/day, although a 3-day cycle may be used. A cycle consists of the dose divided into 3 to 5 equal doses depending upon the schedule fixed. The infusion is given slowly over 4 to 4½ h. During the fusion, vital signs should be monitored.

The initial frequency is generally 1 cycle every 3 to 4 weeks. High-dose i.v. IG is tapered maintaining the same dose but increasing the time interval between infusions. The proposed end point is 2 infusions, each given 16 weeks apart. The cessation of all systemic therapy, including hd i.v. IG, in the absence of clinical disease, is defined as the beginning of the remission period.

## Pretreatment Investigations

Serum levels of immunoglobulins, especially IgA, should be determined. Patients with low or absent levels of IgA may have antibodies to IgA, and such patients develop anaphylaxis. A complete blood cell count, hepatic and renal function tests, and screening for rheumatoid factor and cryoglobulin are recommended. Patients with cryoglobulin have a higher risk to develop acute renal failure. Therapy with hd i.v. IG should be used cautiously in patients with renal insufficiency or impaired cardiac function because fluid overload may occur.

Periodical investigations during treatment consist of complete blood cell count, renal and liver function, and antibodies to HIV, hepatitis A, B and C virus.

## Adverse Effects

Adverse reactions associated with the use of hd i.v IG are usually mild and self limiting.[16] Most adverse reactions will disappear if the infusion is temporarily discontinued or if the infusion rate is lowered. Reactions such as headache, back pain, chills, flushing, fever, hypertension, myalgia, nausea and vomiting appear to be related to infusion rate rather than the dose. Erythema, pain, phlebitis and eczematous dermatitis may occur at the infusion site.

Aseptic meningitis has been reported in patients receiving hd i.v.IG.[17] Stroke and deep vein thrombosis have been reported as complications of hd i.v. IG therapy.[18] Use of hd i.v. IG has been associated with acute renal failure. Patients receiving hd i.v. IG reconstituted from powder products or hd i.v. IG preparations containing sucrose are at a greater risk of renal failure. Jolles *et al.*[17] have tabulated different products available and their constitutes. Since hd i.v. IG is isolated from pooled human plasma, the therapy carries the potential risk of transferring infectious agents. A small sample of i.v. IG should be stored before each infusion for further analysis in the event of infectious disease transmission. The ideal i.v. IG preparation would be sugar free with low sodium content and physiological osmolarity.

Cutaneous adverse events reported with the use of i.v. IG include petechiae, pruritus, urticaria, lichenoid eruptions, alopecia and leukocytoclastic vasculitis.
